# Significant Calcification of an Entire Aortic Tree with Renal Artery Subocclusion: Acute Kidney Injury, Ischemic Kidney Disease, and the Tissue Viability Question—A Case Report

**DOI:** 10.3390/life15010082

**Published:** 2025-01-11

**Authors:** Marko Baralić, Selena Gajić, Aleksandra Kezić, Ana Bontić, Jelena Pavlović, Voin Brković, Vidna Karadžić Ristanović, Danka Bjelić, Maja Životić, Sanja Radojević-Škodrić, Želimir Antonić, Nenad Ilijevski, Milan Radović

**Affiliations:** 1 Clinic of Nephrology, University Clinical Center of Serbia, Pasterova 2, 11000 Belgrade, Serbia; 2 Faculty of Medicine, University of Belgrade, Dr Subotića Starijeg 8, 11000 Belgrade, Serbia; 3 Department of Pathology, Faculty of Medicine, University of Belgrade, Dr Subotića Starijeg 8, 11000 Belgrade, Serbia; 4 Institute of Cardiovascular Disease Dedinje, Department of Radiology, Heroja Milana Tepića 1, 11000 Belgrade, Serbia; 5 Institute of Cardiovascular Disease Dedinje, Clinic of Vascular Surgery, Heroja Milana Tepića 1, 11000 Belgrade, Serbia

**Keywords:** acute kidney injury, hypertension, diabetes mellitus, hemodialysis, histopathological, renal artery stenosis, percutaneous transluminal angioplasty

## Abstract

Background: Undiagnosed and untreated atherosclerotic renal artery stenosis (ARAS) can result in end-stage kidney disease (ESKD). To obtain an accurate diagnosis, it is crucial to recognize the symptoms and signs suggesting renal artery stenosis (RAS) and perform appropriate diagnostic and treatment procedures afterward. Case Presentation: We present a case of a 60-year-old female patient with hypertensive crisis, acute heart failure (HF), and pulmonary edema as the initial signs of acute kidney injury (AKI) caused by right RAS and left renal artery occlusion in the presence of severe aortic atherosclerosis revealed on computed tomography angiography (CTA) of the abdomen. The patient’s renal function recovered completely following percutaneous transluminal angioplasty (PTA) with stent implantation in the right renal artery at the site of subocclusion. Conclusions: Even in patients with concomitant disorders like type-2 diabetes mellitus (T2DM), hypertension (HTN), or HF, the dilatation of significantly narrowed renal arteries due to severe calcifications can result in complete renal function recovery.

## 1. Introduction

Acute kidney diseases and disorders (AKDs) have been defined by abnormalities in kidney function and/or structure present for <3 months and includes acute kidney injury (AKI). AKI has been defined by changes in kidney function, including serum creatinine (Scr) changes and urine output with onset of development within 7 days due to a variety of causes [1]. Chronic kidney disease (CKD) is defined as abnormalities of the kidney structure or function, present for a minimum of 3 months, with implications for health [2]. CKD is one of the most prevalent disorders today, most commonly caused by type-2 diabetes mellitus (T2DM) and hypertension (HTN) [3]. Untreated AKI and CKD can progress to end-stage kidney disease (ESKD) [4,5]. Treatment options include kidney replacement therapy (KRT) with hemodialysis (HD) or peritoneal dialysis (PD) and kidney transplantation [4]. Renovascular disease (RVD) is characterized by the progressive narrowing of the renal arterial or venous vessels, which causes a variety of symptoms ranging from scantly symptomatic disorders and renovascular HTN to prerenal AKI or ischemic nephropathy, resulting in irreversible kidney damage and contributing to CKD. In practice, this term is most commonly associated with renal artery stenosis (RAS), while disorders affecting only the renal veins are unusual [6]. RAS may be caused by numerous pathologies, with fibromuscular dysplasia (FMD) accounting for approximately 10% and atherosclerotic renal artery stenosis (ARAS) accounting for 90% of all renovascular lesions [7]. Classic clinical signs may suggest a diagnosis of RAS, but imaging is necessary to confirm the diagnosis [8]. Hemodynamically significant stenosis is defined as arterial lumen narrowing of more than 70%. Treatment options include optimal medical therapy, such as antihypertensive therapy and guideline-directed therapies to limit atherosclerosis; percutaneous therapy, such as angioplasty and stenting; and surgical therapy, such as aorto-renal bypass surgery, splanchno-renal bypass surgery, and endarterectomy [9,10]. In the most severe cases, kidney auto-transplantation can be used when the above-mentioned methods cannot provide sufficient revascularization [11].

## 2. Clinical Case Presentation

This case study was approved by the Ethical Committee of the University Clinical Centre of Serbia (approval number: 890/8) and conducted following the Declaration of Helsinki and the Ethical Guidelines for Medical and Health Research Involving Human Subjects. Patient informed consent was obtained.

A 60-year-old female patient presented with hypertensive crisis, acute heart failure (HF), and pulmonary edema as the initial signs of AKI. Initial laboratory test results confirmed azotemia with Scr up to 628 μmol/L. She had a Scr of 68 μmol/L three months prior. Anuria and uremia demanded the start of HD treatment. She had a history of comorbid diseases, including T2DM and HTN, and a smoking history of more than three decades. Numerous diagnostic procedures were performed during hospital treatment. Echocardiography revealed a normal result except for mitral regurgitation 2+ and a left ventricle ejection fraction of 56%. From abdominal ultrasonography (US), both kidneys were 120 mm in length and had a parenchyma thickness of 17 mm. An inverted wave was observed above the left renal artery, while the right kidney showed weaker Doppler flow through the renal parenchyma with a resistance index of 0.5. This indicated aortic and renal artery angiography. The biochemistry and hematological analyses from the initial hospitalization are shown in Table 1, while the immunological analyses are given in Table 2.

**Figure 1:**
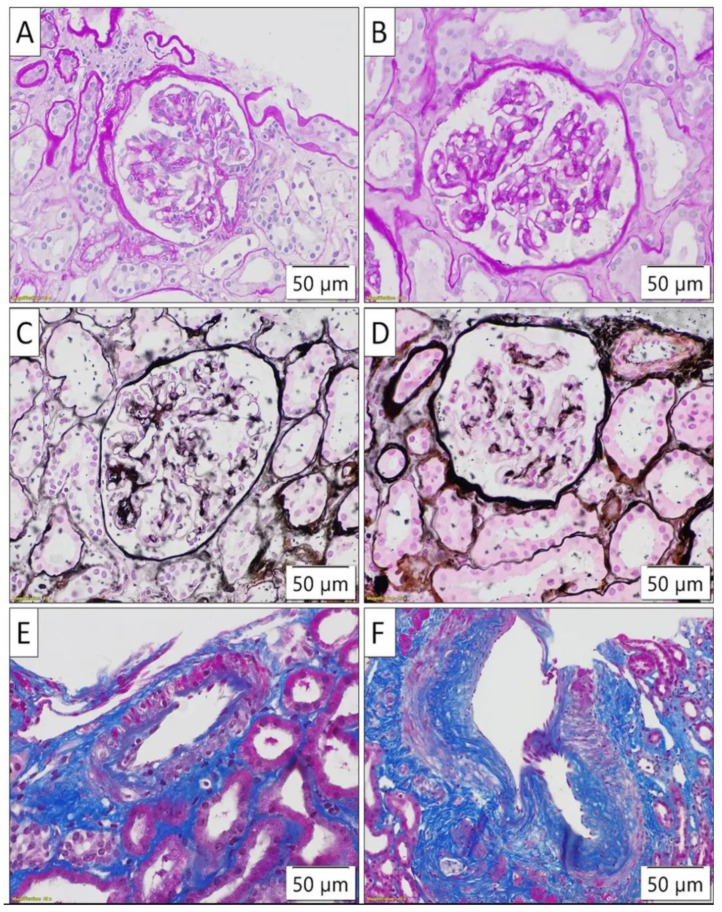
(**A**–**F**) Histopathological findings. The histopathological evaluation of a renal biopsy sample. Subtle changes were observed in the glomeruli, with focal segmental mesangial expansion accompanied by the mild proliferation of mesangial cells (**A**–**D**), as well as focal chronic tubulointerstitial lesions, such as tubular atrophy and interstitial fibrosis, affecting up to 15% of the parenchyma (**A**). The arterioles predominantly exhibited normal morphology (**A**,**D**), while the arteries showed fibrointimal hyperplasia, which was more prominent in larger-caliber blood vessels (**E**,**F**).

Instead, the patient had two of the most common chronic diseases, T2DM and HTN, that can cause CKD, and she had not had microalbuminuria three months prior to hospital admission. So, an ophthalmological examination was performed, which ruled out changes in the blood vessels of the fundus. It was decided that a percutaneous biopsy of the left kidney’s lower pole should be performed under US guidance. All glomeruli had nearly normal histopathological (HP) findings, except a rare mesangium expansion and the proliferation of a few mesangial cells and capsular adhesion in one glomerulus (Figure 1A–D). The tubulointerstitium was minimally changed, with distinct interstitial fibrosis and 15% tubular atrophy (Figure 1A). Myeloelastofibrosis causes thicker blood arteries (Figure 1E,F) without affecting arterioles (Figure 1A,D). The immunofluorescence results were negative. The HP findings corresponded to preserved glomerular and tubular apparatus.

**Figure 2:**
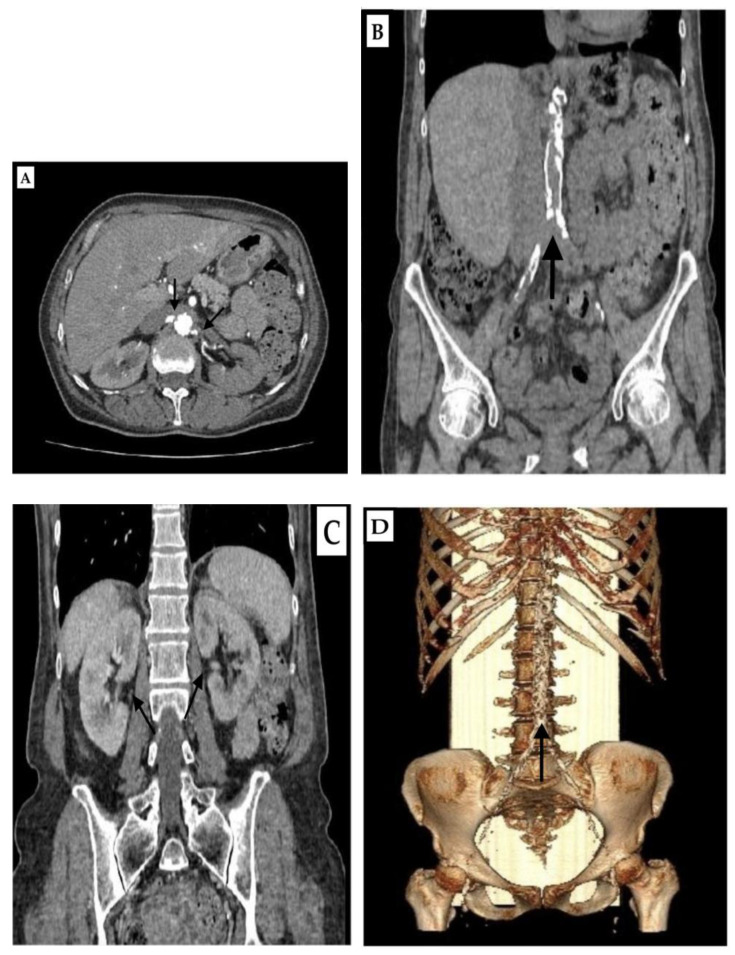
(**A**–**D**) Computed tomography angiography of the abdomen. Axial section, arterial phase, and narrowed lumen at the level of origin of renal arteries (**A**). Native shot, coronal section, and highly calcified aorta (**B**). Coronal section, porto-venous phase, and both kidneys post-contrast-opacified (**C**). Volume rendering (VR) view of a very pronounced calcified aorta (**D**). (Black arrows).

The chest X-ray showed lung emphysema and a 1.3 cm circular shadow in the right perihilar lung field. Computed tomography (CT) pulmoangiography ruled out pulmonary embolism but revealed significant calcification of the thoracic aorta and coronary arteries, centrilobular emphysema with interstitial edema, and pleural effusions with atelectasis of the posterior basal segments. Nelson’s segment of the lower right lobe showed a benign nodular alteration with a diameter of 100 mm, corresponding to a granuloma. The middle lobe had three ground glass nodes with a diameter of 4.7 mm. There were no signs of an active tumor process. Overall, the heart was enlarged. Spirometry suggested mixed ventilatory impairment, predominantly restrictive. Computed tomography angiography (CTA) of the abdomen revealed significant coarse calcifications in the abdominal aorta wall and its branches (Figure 2B), narrowing the lumen to 2 mm at the ostium of both renal arteries (Figure 2A). CT aortorenography confirmed significant right RAS at the ostium and left renal artery occlusion. The committee of vascular surgeons at the institution of the first hospitalization declared that percutaneous transluminal angioplasty (PTA) was impossible due to the high risk of the procedure. Because of the inability to perform PTA, it was determined that renal insufficiency could not be recovered, so we started to prepare the patient for a permanent HD treatment regimen.

**Figure 3:**
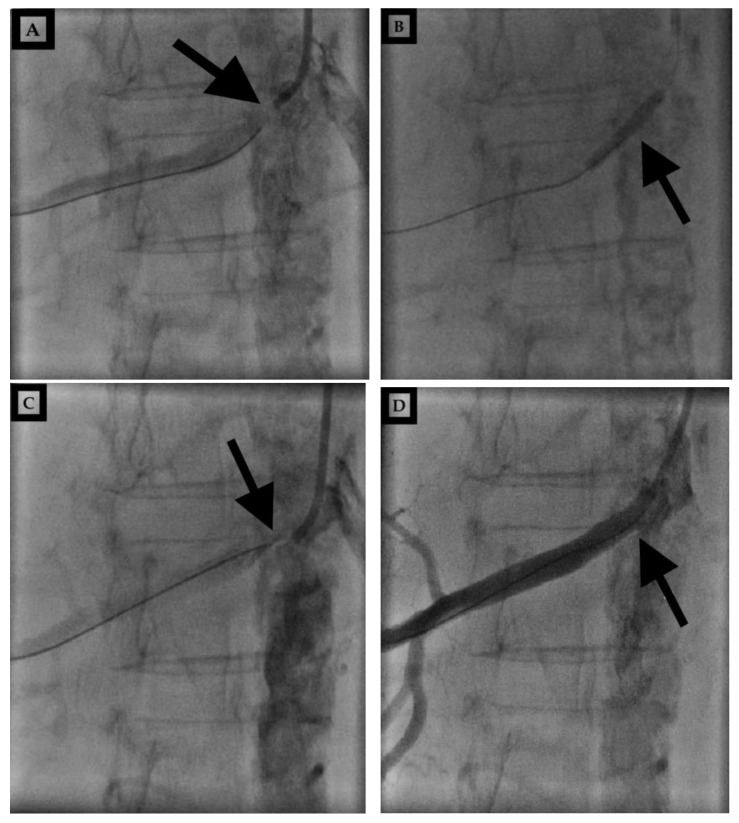
(**A**–**D**) Percutaneous transluminal angioplasty. Native recording before percutaneous transluminal right renal angioplasty and after wire placement (**A**). The presentation of a balloon in the right renal artery (**B**). Marked location after balloon dilation (**C**). Marked stent location and the end final result (**D**). (Black arrows).

The patient sought a second opinion in another institution, where they stated that they could try PTA. An interventional radiologist from the second hospital performed PTA of the right renal artery and implanted a stent at the site of subocclusion (Figure 3), resulting in total renal function recovery. Three months following the PTA procedure, Scr was 60 μmol/L (Table 1). 

**Table 1:** Biochemical and hematological analyses.

Laboratory Values	1st Hospital Admission	1st Day Before PTA	3-Month Follow-Up Period After PTA	Reference Range
Leukocytes 10^9^/L	9.8	9.1	7.4	3.4–9.7
Erythrocytes 10^12^/L	2.16	3.03	-	4.34–5.72
Hematocrit L/L	0.27	0.28	-	0.41–0.53
Hemoglobin g/L	75	91	113	122–157
MCV fL	91	93	-	83.0–97.0
Platels 10^9^/L	96	115	328	150–450
Gly mmol/L	9.8	5.3	7.8	3.9–6.1
Sur mmol/L	17.2	25	6.8	2.5–7.5
Scr μmol/L	462	922	60	45–84
Uric acid μmol/L	288	249	320	210–460
CRP ng/mL	17	12	-	0.0–5.0
Total protein g/L	58	61	65	62–81
Albumin g/L	33	37	35	34–55
Cholesterol mmol/L	4.8	4.4	4.6	0.00–5.20
Triglycerides g/L	1.8	1.9	1.5	0.00–1.70
Ca^2+^ mmol/L	2.15	2.13	-	2.15–2.65
PO_4_^−^ mmol/L	1.3	1.37	-	0.80–1.55
LDH U/L	399	-	-	220–460
Haptoglobin g/L	2.14	-	-	0.30–2.00
Bilirubin umol/L	12.3	-	-	0.0–20.5

MCV—mean corpuscular volume; Gly—glycemia; Sur—urea; Scr—creatinine; CRP—C reactive protein; Ca^2+^—calcium; PO_4_^−^—phosphorus; LDH—lactate dehydrogenase. A hyphen indicates the laboratory value was not measured at the corresponding time.

**Table 2:** Immunological panel.

Immunological Test	Results	Reference Range
ANCA	negative	-
ANA Hep2	negative	-
IgG g/L	8.3	7.65–13.6
IgA g/L	1.99	0.91–2.9
aCL At IgG GPL U/mL	3.1	<10
aCL At IgM MPL U/mL	1.6	<10
C3 g/L	0.97	0.83–2.25
C4 g/L	0.18	0.14–0.35

ANA—antinuclear antibody; ANCA—anti-neutrophil cytoplasmic antibody; IgA and IgG—immunoglobulin A and G; aCL At—anticardiolipin antibodies; GPL—IgG phospholipid; MPL—IgM phospholipid; C3—complement component 3; C4—complement component 4.

## 3. Discussion

Atherosclerosis is the most prevalent etiological cause, accounting for 90% of RAS cases [8,12,13]. ARAS occurs most often in older people due to systemic atherosclerosis and atherosclerotic changes in the abdominal aorta. ARAS is most commonly diagnosed in men (male/female ratio of 2:1) above the age of 50–55 [6]. Renal atherosclerotic plaques are typically bilateral and present as eccentric or concentric lesions approximately 1 cm from the ostium (ostial plaques) or in the proximal one-third of the renal artery [6,7,12,14]. This contrasts with RAS associated with FMD, which often involves the distal two-thirds of the renal arteries [14]. Unlike FMD, atherosclerotic changes in the renal arteries frequently result in the entire occlusion of the renal artery and severe ischemia complications [6]. Our patient had extensive calcifications in the abdominal aorta wall and its branches, as well as significant stenosis at the ostium of the right renal artery and left renal artery occlusion due to coarse calcifications.

Patients with ARAS frequently have risk factors such as T2DM, HTN, dyslipidemia, a smoking history, peripheral vascular disease, and coronary syndrome [6]. Our patient had a long-term smoking history, T2DM, and HTN. RAS with hemodynamically significant narrowing of the renal artery can cause three clinical problems, renovascular HTN, ischemic nephropathy, and cardiac destabilization syndrome [7,13,15], as a result of cardio-renal syndrome with renin–angiotensin–aldosterone (RAAS) system activation [16]. Cardiac destabilization syndrome includes acute decompensated HF and acute coronary syndromes (ACSs) [13]. Acute decompensated HF, or flash pulmonary edema, usually occurs with bilateral RAS or RAS affecting a solitary functioning kidney [14].

Therefore, the clinical clues that suggest the diagnosis of RAS include hypokalemia, the presence of an abdominal bruit and the onset of HTN before 30 years of age, or the onset of severe HTN after 55 years of age or onset in the absence of a family history of HTN; accelerated HTN (sudden, persistent worsening of previously controlled HTN); resistant HTN (HTN not controlled with a three-drug regimen that includes a diuretic) and malignant HTN (HTN with acute end-organ damage); new onset renal dysfunction or the worsening of renal function after starting therapy with an angiotensin-converting enzyme inhibitor (ACEI) or angiotensin receptor blocker (ARB); an unexplained atrophic kidney or discrepancy in the size of the two kidneys > 1.5 cm; and sudden, unexplained pulmonary edema, especially in patients with azotemia [8,9]. Our 60-year-old female patient was admitted to the hospital with a blood pressure of 210/120 mmHg, acute HF with NT-proBNP 4067 pg/mL, pulmonary edema, and AKI with anuria and an increase in Scr from 68 to 628 μmol/L in less than 3 months.

When RAS is suspected, imaging is used to confirm the diagnosis [6,8,13,14]. Artery angiography is the diagnostic gold standard for RAS [6,14,17,18]. It has good picture quality and allows pressure gradients across the stenosis to be measured during endovascular treatment. However, compared to cross-sectional imaging, it is more invasive. It has a higher risk of complications, such as vascular complications at the puncture site, atheroembolic events, allergic reactions to contrast, and contrast-induced nephropathy. Arteria angiography provides higher levels of contrast and radiation dosage [17]. Therefore, arteria angiography should not be used as an initial diagnostic test [6,14,17], but it should rather be used after a positive noninvasive screening test or in cases where RAS is strongly suspected and definitive noninvasive imaging is unavailable or inconclusive [7,13,18]. Captopril renography, Doppler US, CTA, and magnetic resonance angiography (MRA) are noninvasive imaging procedures for diagnosing RAS [14,18]. Doppler US, which combines the direct viewing of renal arteries and Doppler velocity measures of blood flow, is an excellent screening test for RAS due to its low cost, accessibility, non-toxicity, and lack of ionizing radiation exposure [6,8,14]. However, the US is limited by abdominal fat or intestinal gas, has low sensitivity, and depends on the operator’s skill [8,14,17]. CTA and MRA are more accurate than the US [6] but have several disadvantages. CTA exposes patients to radiation and iodine contrast. Intravascular contrast can cause allergic reactions and contrast-induced nephropathy. MRA does not expose the patient to radiation or iodine contrast, but patients with reduced renal function may develop nephrogenic systemic fibrosis after using gadolinium-based contrast. MRA may not be possible due to claustrophobia and MRA-incompatible implantable devices [14]. 

Based on the clinical presentation, we suspected RAS, but we performed additional tests to rule out other potential causes of AKI. Our patient was hypervolemic when admitted but became euvolemic after starting HD. She had no history of using nephrotoxic drugs. The US revealed normal kidneys without stasis or calculosis. We could not perform urine sediment and 24 h proteinuria since the patient was anuric upon admission. Although the ADAMTS13 test was not performed for technical reasons, considering hemolysis parameters, haptoglobin, LDH, and bilirubin were within the reference range, and the suspicion of thrombotic microangiopathy (TMA) was excluded. Anemia was managed with two doses of erythrocyte transfusion, and once the blood pressure was normalized, the platelet count was restored spontaneously. The immunology panel and kidney biopsy ruled out glomerulonephritis. Doppler US revealed an inverted wave above the left renal artery, while the right kidney had weaker flow through the parenchyma with a resistance index of 0.5. Because of AKI, CTA was challenging to perform due to contrast nephropathy. However, due to the high suspicion of RAS and the significance of accurate diagnosis and treatment, we performed CTA and provided the patient with HD after the procedure. CTA detected RSA and CT aortorenography confirmed severe right RAS at the ostium and left renal artery occlusion.

All ARAS patients should receive appropriate medical therapy, including HTN control, T2DM control, statins, antiplatelet medication, smoking cessation, and an increase in physical activity [10]. The indications for revascularization are unclear, and the available randomized controlled trials have limitations [6,14]. Patients in randomized control studies had low-risk ARAS with <70% stenosis and normal or mildly reduced renal function. The selection of low-risk participants had an impact on the trial results. A sub-analysis of the trials found that participants with an ARAS of 80% or higher, acute decompensated HF, unstable angina, rapid deterioration of renal function, and severe uncontrolled HTN significantly benefited from renal revascularization in addition to optimal medical therapy [13]. According to the European Society of Cardiology’s 2017 guidelines, revascularization should be considered in patients with ARAS and unexplained pulmonary edema or HF [14]. The Society for Cardiovascular Angiography and Interventions released an appropriate use criteria statement in 2018 for renal artery stenting. They reported that stenting was a suitable treatment for persons with severe ARAS and flash pulmonary edema, bilateral ARAS, or a single viable kidney with ARAS and declining renal function. Stenting may be appropriate for patients with severe ARAS and failure to control blood pressure on three maximally treated medications, one of which is a diuretic, as well as the following: severe ARAS and recurrent acute decompensated HF requiring hospitalization despite being on maximal medical treatment; unilateral severe ARAS with declining renal function; and severe ARAS with acute coronary syndrome while on optimal medical therapy [13]. Renal artery angioplasty and stenting are currently preferred over open revascularization [14]. Stenting is associated with higher technical success and lower restenosis than balloon angioplasty alone [6,12,14]. Open surgery is used when percutaneous interventions fail for patients with complex renal artery anatomy or for those who require open surgery for aortic diseases such as an aneurysm [14].

Vascular surgeons concluded during the initial hospitalization that PTA was impossible to perform due to the significant calcification of the aorta and its branches, as well as the procedure’s high risk due to patient comorbidities. Given that the kidney’s HP was normal, the patient requested a second opinion in another institution, where an interventional radiologist performed PTA of the right renal artery and placed a stent at the site of subocclusion. This procedure was performed with appropriate nephrological preparation. Renal function continually improved, and two additional HD treatments were performed following PTA.

The function of the remaining kidney was then recovered to a normal level, and the patient had normal Scr levels for three months throughout subsequent nephrological checks. The interesting and still debatable issue is the influence of ischemic time on the choice of treatment, specifically the decision regarding surgical or interventional options. According to guidelines, revascularization or percutaneous endovascular therapy such as thrombectomy, stent placement, or thrombolysis are advised if the occlusion time is less than 6 h [19,20]. However, there are some reports of successful renal recovery after these interventional therapies performed even after 14 days and up to 3 months of persistent renal artery occlusion [21]. The presence of renal collateral circulation via capsular and peripelvic branches can explain renal viability during renal artery occlusion and the successful recovery of renal function after the restoration of renal circulation [22]. HP findings of the kidney specimen in our patient were a confirmatory sign of the viability of renal parenchyma despite complete renal artery occlusion.

## 4. Conclusions

The dilatation of significantly narrowed arteries caused by extensive calcifications can result in complete renal function recovery, even if the patient has associated diseases such as T2DM, HTN, or HF.

Percutaneous renal artery dilatation with stenting is one treatment option for patients with renal artery occlusion, even in prolonged ischemia, measured by days or weeks, but evidence of the viability of renal parenchyma is needed. The recovery of renal function can be expected up to the Scr value that the patient had within three months before the development of AKI.

## Data Availability

The original contributions presented in this study are included in the article; further inquiries can be directed to the corresponding author.
